# Intramedullary nailing appears to be superior in pertrochanteric hip fractures with a detached greater trochanter: 311 consecutive patients followed for 1 year

**DOI:** 10.3109/17453674.2011.566143

**Published:** 2011-04-05

**Authors:** Henrik Palm, Charlotte Lysén, Michael Krasheninnikoff, Kim Holck, Steffen Jacobsen, Peter Gebuhr

**Affiliations:** Department of Orthopaedic Surgery, Hvidovre University Hospital, Copenhagen, Denmark

## Abstract

**Background and purpose:**

In recent years, intramedullary nails (INs) for the treatment of pertrochanteric hip fractures have gained prominence relative to conventional, sliding hip screws (SHSs). There is little empirical background for this development, however. A previous series of ours suggested that the use of SHS was not adequate in situations with fragile or fractured lateral femoral walls, where it often led to lack of healing in a maximally telescoped position. We hypothesized that INs would be the superior implant in these specific circumstances.

**Methods:**

We retrospectively examined 311 consecutive patients treated in our department between 2002 and 2008, with either an IN (n = 158) or an SHS (n = 153) mounted on a 4-hole side-plate, for an AO/OTA type 31A1–2 pertrochanteric fracture with a detached greater trochanter. The status of the lesser trochanter was assessed preoperatively and the integrity of the lateral femoral wall, fracture reduction, and position of the implants were assessed postoperatively. Reoperations due to technical failure were recorded for one year postoperatively.

**Results:**

Multivariate logistic regression analysis showed that the groups were similar regarding demographic and biomechanical parameters. The lateral femoral wall was more frequently fractured during SHS implantation (42 patients) than in the IN group (9 patients) (p < 0.001). 6 (4%) of the 158 patients operated with IN had to be reoperated, as compared to 22 (14%) in the SHS group of 153 patients (p = 0.001).

**Interpretation:**

IN had a lower reoperation rate than SHS in these pertrochanteric hip fractures with a detached greater trochanter. IN left more lateral femoral walls intact.

In the last decade, the use of intramedullary nails (INs) for the treatment of pertrochanteric fractures has become more common (Figure 1). There is little empirical background for the development, however (Stern 2007, Anglen and Weinstein 2008, Parker and Handoll 2008, Rogmark et al. 2010). The latest Cochrane review recommends conventional sliding hip screws (SHSs) for these fractures, but also recommends more studies to determine whether INs are of advantage for selected fracture types (Parker and Handoll 2008). In a randomized study, Saudan et al. (2002) found no advantages using INs for the AO/OTA type A1 and A2 fractures as a whole, but to our knowledge no studies comparing the rate of reoperation in the AO/OTA type A1 and A2 subgroups have been published.

**Figure 1.**
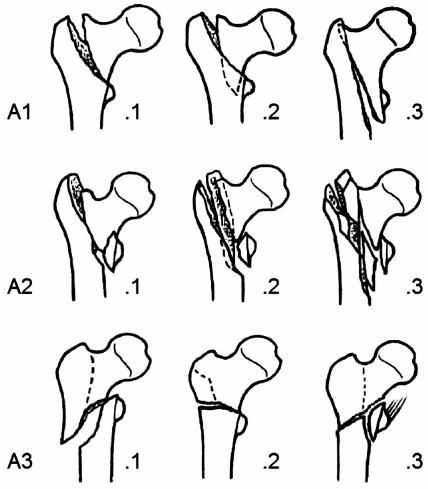
AO/OTA type-31-A pertrochanteric fractures. 31-A1 fractures are simple, whereas 31-A2 fractures are multifragmentary. Subgroups of 31-A2 pertrochanteric fractures are A2.1 (detachment of the lesser trochanter), A2.2 (several intermediate fragments including detachment of the lesser trochanter), and A2.3 (several intermediate fragments extending more than 1 cm distal to the lesser trochanter). 31-A3 intertrochanteric fractures all have a fracture line through the lateral femoral wall, anatomically defined as the lateral femoral cortex distal to the greater trochanter. (Reprinted, with permission from: Orthopaedic Trauma Association Classification, Database and Outcomes Committee. Fracture and dislocation classification compendium - 2007. J Orthop Trauma 2007; 10 Suppl.)

Recently, we performed a cohort study that indicated that SHS is not adequate in patients with fragile lateral femoral walls (Palm et al. 2007). We found that this important lateral buttress area was fractured during the operative procedure in a third of patients with a detached greater trochanter preoperatively (Figure 2).

**Figure 2.**
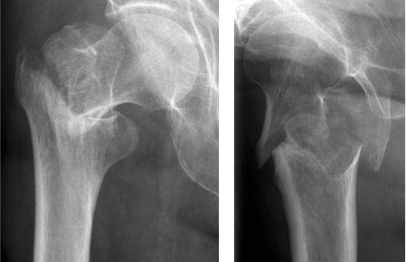
An 82-year-old woman sustained a pertrochanteric hip fracture with a detached greater trochanter.

We have now compared the rate of reoperations in the subgroup of pertrochanteric hip fractures with a detached greater trochanter treated either with IN or SHS. In particular, we wanted to assess whether there was a risk that the implants would cause a fracture of the lateral femoral wall.

## Patients and methods

The 635 consecutive patients admitted to our hospital between September 2002 and July 2008 were prospectively included in a database after having sustained a pertrochanteric AO/OTA type A1 or A2 fracture treated with either (1) a 130° short intramedullary nail (IMHS; Smith and Nephew, Memphis, TN) inserted antegrade without reaming or use of circulating wires, or (2) a sliding hip screw mounted on a 135° 4-hole side-plate (HipLOC; Biomet, Warsaw, IN). The senior surgeon on duty chose the type of surgery. One author (HP) retrospectively assessed the status of the greater trochanter on preoperative radiographs. An intra- and interobserver study of this was performed twice by HP and KH on 100 randomly selected patients, 3 weeks apart. At the time of assessment, the observers were blind as to the type of operation and as to which patients later required a reoperation. The agreement showed a kappa value for interobserver reliability of 0.76 (0.70–0.83) and intraobserver reliability varying from 0.80 (0.74–0.86) for HP and 0.82 (0.76–0.88) for KH. 314 patients were found to have a detached greater trochanter. As 3 patients died of unrelated causes before the postoperative radiographic examination, 311 patients were entered into the study.

The study was part of the hip fracture project at Hvidovre University Hospital, Copenhagen, Denmark. It was approved by the Danish data protection agency and Copenhagen ethics committee, which determined that the nature of the study was such that written patient consent would not be required.

All patients were managed with the department's specialized fast-track protocol for hip fractures (Foss et al. 2005). The patients were operated during daytime with epidural anesthesia. Preoperatively, a single dose of 1.5 g cephalosporin was administered intravenously. Postoperatively, low-molecular-weight heparin was administered until the patient was fully mobilized, but for a minimum of 5 days. Mobilization was encouraged, starting on the day of surgery, in an intensive physiotherapy program.

The patients were given the American Society of Anesthesiologists (ASA) physical grading score (a scale of 0 to 4) (American Society of Anesthesiologists 1963) and the Parker New Mobility score (NMS, a scale of 0 to 9 with ≤ 5 designating an inhibited functional level) (Parker and Palmer 1993). In all patients, radiographs (anterior-posterior and lateral) were obtained preoperatively and postoperatively within the first 3 days. These images were used to assess the status of the greater and lesser trochanters and to classify the fractures according to the AO/OTA classification preoperatively (Figure 1) and the integrity of the lateral femoral wall postoperatively. The position of the implants (tip-apex distance) was determined according to the method of Baumgaertner et al. (1995). Resultant fracture reductions were measured in mm on both the anteroposterior and the lateral radiographs, and summarized.

The rate of reoperation within 1 year was registered from patient records and cross-checked with the Copenhagen radiographic database. Reoperations for technical reasons were registered as outcome parameter. The department's guidelines on indications for reoperation for technical reasons were: (1) subsequent fracture around the implant, (2) cut-out of the screw from the femoral head into the hip joint, (3) progressive fracture displacement, defined as a displaced but still unhealed fracture in combination with progressive migration and/or maximum shortening of the screw in the femoral head, (4) loosening of the SHS plate, or if (5) the distal locking screws were placed outside the IN.

### Statistics

Differences in demographic and clinical parameters were analyzed using chi-square tests for dichotomized data and the Mann-Whitney test for linear data. Demographic and clinical parameters that might hypothetically influence the rate of reoperation were entered into multivariate regression analyses. Intra- and interobserver reliability was calculated by the Cohen kappa. Patient survival between the 2 groups was analyzed using Kaplan-Meier survival tables. The level of significance was set at p < 0.05. All calculations were performed using SPSS statistical software version 16.0.

## Results

Age, sex, NMS, ASA score, and fracture reduction were similar in the 2 groups (Table 1). However, the tip-apex distance was statistically significantly lower, and detachment of the lesser trochanter was seen statistically significantly more often, in patients who had an IN inserted. 36 (12%) of the 311 patients included were reoperated within a year. 2 of these patients had surgical drainage of a hematoma and 4 were reoperated because of infection. 1 patient had a total hip replacement due to severe osteoarthritis and 1 patient had the implant removed after the fracture had healed. All but the last patient had an SHS performed primarily. In the remaining 28 (9%) of the 311 patients, the reoperation was performed because of technical failure.

**Table 1.** Data on the 311 patients with a pertrochanteric fracture with a detached greater trochanter, operated with either an intramedullary nail or a sliding hip screw

	Intramedullary nail n (%)	Sliding hip screw n (%)	p-value
No. of patients	158 (100)	153 (100)	
Age, years **^a^**	84 (75–90)	83 (76–90)	0.7
Female sex	120 (76)	120 (78)	0.6
Prefracture NMS 0–5	73 (47)	78 (51)	0.5
ASA score III–IV	66 (42)	75 (49)	0.2
Detached lesser trochanter	132 (84)	85 (56)	< 0.001
Tip-apex distance, mm **^a^**	20 (15–25)	21 (16–27)	0.02
Fracture reduction, mm **^a^**	7 (2–11)	8 (4–14)	0.1
Reoperation within a year	6 (4)	22 (14)	0.001
Screw cut-out through femoral head	3 (2)	13 (8)	
Progressive fracture displacement	1 (< 1)	8 (5)	
Subsequent fracture around the implant	1 (< 1)	1 (< 1)	
Distal locking screws outside the nail	1 (< 1)	– (–)	

ASA: American Society of Anaesthesiologists; NMS: new mobility score. Values are presented as number of patients (percentage) and analyzed using the chi-square test, **^a^** except for continuous data, presented as median (interquartiles) and analyzed using the Mann-Whitney test.

14% of the 153 patients with an SHS and 4% of the 158 patients with an IN were reoperated due to technical failure within 1 year (p = 0.001) (Table 1). In both groups, screw cut-out was the main reason for reoperation. In a multivariate logistic regression analysis combining age, sex, ASA score, NMS, status of the lesser trochanter, fracture reduction, and implant position, SHS was the main independent risk factor for reoperation (p = 0.002). The fracture reduction was also found to have an independent effect on the outcome (Table 2).

**Table 2.** Relationship between reoperation within a year postoperatively and patient characteristics in the 311 patients with a pertrochanteric fracture with a detached greater trochanter, operated with either an intramedullary nail or a sliding hip screw

	n (%)	Reoperation within 1 year postoperatively			
		Univariate analysis		Multivariate analysis	
		OR (95% CI)	p-value	OR (95% CI)	p-value
Age, years **^a^**	84 (76–90)		1.0	1.0 (1.0–1.1)	0.2
Female sex	240 (77)	0.6 0.3–1.4)	0.2	0.5 (0.2–1.4)	0.2
Prefracture NMS 0–5	151 (49)	0.7 (0.3–1.5)	0.4	0.5 (0.2–1.4)	0.2
ASA score III–IV	141 (46)	1.1 (0.5–2.5)	0.8	1.2 (0.5–3.0)	0.7
Detached lesser trochanter	217 (70)	0.9 (0.4–2.1)	0.8	1.6 (0.6–4.0)	0.4
Tip-apex distance, mm **^a^**	20 (15–26)		0.09	1.1 (0.9–1.1)	0.07
Fracture reduction, mm **^a^**	8 (3–12)		0.02	1.1 (1.0–1.1)	0.01
Sliding hip screw	153 (49)	4.3 (1.7–11)	0.001	5.3 (1.8–15)	0.002

For abbreviations and legend, see Table 1

Preoperatively, all fractures had an intact lateral femoral wall, as the inclusion criterion was patients with an AO/OTA type A1 or A2 pertrochanteric fracture. Postoperatively, the lateral femoral wall was fractured in 28% (42/153) of patients with an SHS and in 6% (9/158) of patients with an IN (p < 0.001). Among the SHS patients, a fractured lateral femoral wall was found to be a predictor of reoperation (31% (13/42 fractured) vs. 8% (9/111 unfractured), p < 0.001).

The median (interquartile range) blood loss intraoperatively was higher in patients with IN than in patients with SHS: 250 (200–350) mL vs. 200 (100–300) mL (p = 0.004). Also, the median skin-to-skin time was longer for patients with IN than for patients with SHS: 68 (56–85) min vs. 59 (41–70) (p < 0.001). 27% (84/311) of the patients died within the first postoperative year, with no significant difference in average number of days (95% CI) of survival between the two groups: 283 (262–304) days for patients with IN vs. 293 (273–314) days for patients with SHS (p = 0.3).

## Discussion

Our reoperation rate at 9% is slightly higher than that in other studies (Stern 2007, Anglen and Weinstein 2008, Parker and Handoll 2008), which can be explained by all the fractures in the present study having a detached greater trochanter. In this subgroup, we—in contrast to all other studies performed on the main group of pertrochanteric fractures—found a 4 times higher risk of reoperation when using the SHS. In both groups, most of the reoperations were caused by progressive fracture displacement and screw cut-out.

Part of the explanation for these failures appears to be that the lateral femoral wall often fractures during insertion of the SHS, thereby worsening the AO/OTA type A2 pertrochanteric fracture into a more unstable AO/OTA type A3 intertrochanteric fracture (Palm et al. 2007). We believe that although using a protractor when inserting the SHS, the proximal part of the fragile lateral femoral wall is forced outwards when tightening the cortical screws in the fixed-angle plate, until a fracture occurs through the large drilled hole for the implant. The IN appears to be superior by maintaining the integrity of the lateral femoral wall. The reason could be that the nail-screw angle is fixed through the guide system, and if the lateral wall is fractured, the nail itself could have a lateral buttress effect by direct contact of the proximal part of the nail with the neck-head fragment.

The lower reoperation rate for IN was found in a multivariate analysis including biomechanical confounders (implant position and fracture reduction), and despite the fact that more IN patients had a detached lesser trochanter. The superior tip-apex distance in the IN group did tend to reduce the reoperation rate in the multivariate analysis, but we believe that using the guide system for the IN reduces the tip-apex distance, especially in the lateral radiographs. As that guide system is only part of the IN procedure, the method of nailing will in fact enable the surgeon to achieve an improved implant position and thereby a reduced risk of reoperation. The overall outcome was in fact worse in the group of patients with an SHS, as we chose to exclude the non-technical reasons for reoperation, because they could be influenced by factors not includable in the regression analysis. As in other studies (Stern 2007, Anglen and Weinstein 2008, Parker and Handoll 2008), these biomechanical advantages were achieved without any increased mortality in the IN group of patients. The IN procedure did, however, result in more intraoperative bleeding and—less importantly, as we would rather do it correctly than quickly—an average of 9 min longer skin-to-skin time. Theoretically, IN is a less traumatic minimally invasive procedure than SHS, and the longer skin time and higher blood loss in the IN group might be explained by the fact that more IN patients had a detached lesser trochanter.

To our knowledge, no other studies have so far shown such a difference in reoperation rate between the two types of implants (Saudan et al. 2002, Stern 2007, Anglen and Weinstein 2008, Parker and Handoll 2008). The latest Cochrane review includes 36 randomized studies comparing IN and SHS, 4 of which were performed on the same two implants as in our study. Here, additional studies are required, as no overall difference in rate of reoperation could be identified (Parker and Handoll 2008).

Some previous studies—especially of the early, more curved nails—have indicated that using an IN increases the risk of shaft fracture (Parker and Handoll 2008). We only experienced one such fracture in our study, and we believe that a detached greater trochanter might diminish the risk. A fractured entry point zone could theoretically offer the surgeon a more flexible entry point, as the bone pieces can adjust to the proximal part of the nail, thereby reducing the need for femoral bending around the distal part of the nail, which reduces the bone stress around the distal locking screw holes.

To our knowledge, all previous randomized studies have (as a minimum) included the main group of AO/OTA type A1 and A2 fractures (Saudan et al. 2002, Parker and Handoll 2008) and could therefore have missed a difference in the subgroup of pertrochanteric fractures with a detached greater trochanter that we analyzed. Also, in our database material the reduced reoperation rate for the IN would have been missed in the main group of 635 patients with an AO/OTA type A1 or A2 pertrochanteric fracture with or without detachment of the greater trochanter. Of the 164 patients operated with an IN, 4% (6) were reoperated—as compared to 6% (30) of the 471 patients operated with an SHS (p = 0.2). Had we not sub-classified our patients, we would not have found the lower reoperation rate in patients with an IN in the subgroup of pertrochanteric fractures with a detached greater trochanter. The reliability when classifying into subgroups is poor (Embden et al. 2010), but we found a substantial agreement when only sub-classifying the AO/OTA type A1 or A2 pertrochanteric fractures into 2 groups based on detachment of the greater trochanter in both the anterior-posterior and the lateral radiographs.

So far, we have classified all our pertrochanteric fractures with a detached greater trochanter as AO/OTA type A2.2 and A2.3 fractures. How should a pertrochanteric hip fracture with a detached greater trochanter and an intact lesser trochanter—like the one presented in Figure 2—really be classified? With 3 fragments, it cannot be classified as an AO/OTA type A1 fracture, but when the lesser trochanter is intact, it is actually not an AO/OTA type A2 fracture either. As the greater trochanter appears to be the key element for choice of implant, we find that a more precise classification is necessary.

Our study is a retrospective cohort study, and some randomized trials based on this subgroup of fractures should be performed. Other types of IN and SHS should be compared, and important parameters that were not available in our database need attention, such as activities of daily living, pain, and mobility. We consider it important that future randomized trails should classify trochanteric fractures into specific subgroups when comparing operative procedures, although the number of patients needed would be higher. At the moment, we recommend using a short intramedullary nail if the lateral wall seems fragile or the greater trochanter is detached.
